# School Girls

**Published:** 1871-09

**Authors:** 


### SCHOOL GIRLS,

Every one of them, high and low, rich and poor, especially the ugly ones, should be taught to consider that their education would be sadly neglected, would not merit a diploma, until they had learned two branches, learned them thoroughly, effectually, perfectly. If six months of the time of the "course" were given to each, there would be a result, moral, social, pecuniary, not easily expressed by figures- One of these branches is partially taught in some schools; if thoroughly in any, the knowledge of such a school should be published throughout the length and breadth of the land.

If such institutions as have been established by the liberality and far-seeing wisdom of such honored names as Matthew Vassar, and Major George Sibley, and his loving wife Mary E., the former on the banks of the glorious Hudson, the latter on the turbid Missouri, far, far away, they would be pioneers in a work which would merit them a brighter immortality than belongs to any name of mere conquering hero. These important branches are —

SEWING AND COOKING, or the pot and the needle. In other words there ought to be a kitchen and a tailor's shop attached to every institution of learning intended for the education of girls, for that sphere of life in which they all expect to move, and of which they are to become the queens, — the household, the fireside, the family circle.

It is not necessary that every wife should do the sewing and cooking of the family; but every wife ought to know how these should be done, so as to be able to make those who do her work do it properly, which would secure an amount of comfort to any family not easily computed. Now and then a wife may be in a pecuniary condition which exempts her from the drudges of the needle and the kitchen, and every mother and every marriageable daughter has an undefined hope or expectation that she is to marry "rich enough" to have these burdens rolled on other shoulders; but it ought to be remembered, that of any thousand girls who get husbands, nine hundred and ninety-nine perhaps will have to do, sooner or later, more or less of their own needle and kitchen work.

On the other hand, not one man in a thousand deserves to have a wife who has the strength of mind, the moral courage, the force of character which prompt her thus to prepare for life's duties, because nine men out of ten — ninety-nine out of a hundred — would prefer the woman who had the most money to her who could make the best shirt or could bake the best loaf of bread.

On Murray Hill there lives a girl without a portion, educated in one of the best schools in New York. She was one of the very best scholars in that school, of worthy parentage, for her father was a good man, and honest until the day of his death ; her mother a lady, every inch of her. Our heroine is a Christian woman, a Church worker ; her hand is in every good thing. She does not play on the piano, she does not sing ; but she has learned from her mother to be the tidiest of housekeepers ; she was learned early to market for the family ; there is not a dish that she cannot go into the kitchen and prepare, not a pie or a cake that she does not know all about ; she makes and fits her own dresses, and trims her own bonnets ; hems her own handkerchiefs ; and seams, and gores, and furbelows, and flounces are A B C to her. Delicate in her tastes, queenly in her manners, faultless in form, and beautiful withal, she would be a treasure to any millionaire or nobleman. But she is poor, unobtrusive, and is rapidly approaching that age which will entitle her to the unenvied designation of

AN OLD MAID.

Shame, a burning shame to the perspicacity of the many young gentlemen of New York, who for want of the restraining influences of a good wife are verging towards bachelorism, club-life, the racecourse, the theatre, and the dram-shop.

Small encouragement indeed is there in this country for an enterprise lately undertaken in London, by a young lady, who has opened an institution for the training of young women to the practice of household duties and of domestic economy, and is encouraged in the same by the entrance of pupils from the families of noblemen, bankers, merchants, and others, whom it is not likely that there ever will be any necessity for their practicing what they are about to learn. But in our country, where fortunes seldom pass to grandchildren, and are often exhausted long before the children become gray-headed, such schools should be universally patronized, so that in case riches should take wings and fly away, they could turn their hands to the utilities of life, and save themselves from starvation and the street.

